# AI-Assisted CT as a Clinical and Research Tool for COVID-19

**DOI:** 10.3389/frai.2021.590189

**Published:** 2021-07-20

**Authors:** Zion Tsz Ho Tse, Sierra Hovet, Hongliang Ren, Tristan Barrett, Sheng Xu, Baris Turkbey, Bradford J. Wood

**Affiliations:** ^1^ Department of Electronic Engineering, The University of York, York, United Kingdom; ^2^ Department of Electronic Engineering, The Chinese University of Hong Kong, Hong Kong, China; ^3^ Department of Radiology, University of Cambridge School of Clinical Medicine, Cambridge, United Kingdom; ^4^ Center for Interventional Oncology, Radiology and Imaging Sciences, National Institutes of Health, Bethesda, MD, United States

**Keywords:** COVID-19, computed tomography, RT-PCR, artificial intelligence, diagnosis

## Abstract

There is compelling support for widening the role of computed tomography (CT) for COVID-19 in clinical and research scenarios. Reverse transcription polymerase chain reaction (RT-PCR) testing, the gold standard for COVID-19 diagnosis, has two potential weaknesses: the delay in obtaining results and the possibility of RT-PCR test kits running out when demand spikes or being unavailable altogether. This perspective article discusses the potential use of CT in conjunction with RT-PCR in hospitals lacking sufficient access to RT-PCR test kits. The precedent for this approach is discussed based on the use of CT for COVID-19 diagnosis and screening in the United Kingdom and China. The hurdles and challenges are presented, which need addressing prior to realization of the potential roles for CT artificial intelligence (AI). The potential roles include a more accurate clinical classification, characterization for research roles and mechanisms, and informing clinical trial response criteria as a surrogate for clinical outcomes.

## Introduction

Computed tomography (CT) is not being used to its full potential toward a better understanding of COVID-19. The reverse transcription polymerase chain reaction (RT-PCR) test is the current gold standard for diagnosing COVID-19 through the detection of nucleic acid present in SARS-CoV-2, the virus that causes COVID-19 (Ai et al., 2020). One of the main disadvantages with RT-PCR testing is that the results may take several hours to several days to obtain. Another disadvantage is that RT-PCR test kits are a finite resource. In many instances, test kits have become temporarily limited in areas experiencing outbreaks as the demand has spiked. Moreover, in low- and middle-income countries, it has been a struggle to access test kits altogether or in the quantity needed (Peplow, 2020). Any hospital equipped with a CT scanner could potentially use CT in conjunction with RT-PCR to address these problems. In specific high-prevalence settings early in the pandemic, the sensitivity of chest CT for detecting COVID-19 has been reported as high as 97–98%, compared to 71–85% for early real-time reverse transcriptase polymerase chain reaction (rRT-PCR) (Ai et al., 2020; Bernheim et al., 2020; Kanne, 2020; Xie et al., 2020). There has been much debate over the ability to generalize such high performance numbers, which are highly dependent on background prevalence in the community or screening population, clinical suspicion, and the performance of specific RT-PCR methodologies, which has improved over the past year.

To complement the use of standard chest CT for COVID-19 characterization and to further contribute to the body of knowledge for combating the pandemic, artificial intelligence (AI) could be utilized to improve the ability of CT to swiftly and accurately flag CTs for immediate interpretation, characterize COVID-19 for clinical research, such as response to medical countermeasures, and potentially increase patient safety by optimizing radiation exposure.

## The Role of CT in the COVID-19 Pandemic

### Using CT in Conjunction With RT-PCR for COVID-19 Diagnosis

To address the aforementioned weaknesses of RT-PCR (namely, the delay in results and fluctuating availability), CT could be used in conjunction with RT-PCR in selected target populations with a high risk for COVID-19, such as at the point of care (POC) in an outbreak setting. In such a POC setting, CT could be used to identify possible cases, and RT-PCR would be used to confirm conclusive diagnosis. CT might solve the problem of delayed results because CT results with or without AI models are obtained more quickly compared to RT-PCR. CT scanners are widely available and offer reliable daily accessibility. Moreover, CT has been shown to assist with detecting possible cases of COVID-19, even among asymptomatic patients, which may be important given the public health conundrum of presymptomatic and asymptomatic transmission (Apostolopoulos and Bessiana, 2003; Narin et al., 2003; Wang and Wong, 2003; Bernheim et al., 2020; Kanne, 2020; Liang, 2020; Xie et al., 2020). Some studies have touted the sensitivity of chest CT (vs. RT-PCR) for detecting COVID-19 in super-acute high-prevalence early epidemic outbreak settings (Ai et al., 2020; Liu et al., 2020). Another study found that RT-PCR and CT together may miss fewer patients than CT alone or RT-PCR alone (Inui et al., 2020).

Certain high-risk patients who undergo CT in an outbreak setting or after a high dose exposure might be identified by CT and isolated or quarantined while they await RT-PCR results, thus reducing the risk of transmission (Amalou et al., 2020). CT scans for other indications, performed in asymptomatic patients, might also identify and isolate patients prior to risking transmission to a busy clinic POC setting. This is important because many people infected with SARS-CoV-2 have mild symptoms or no symptoms at all. Additionally, peak viral shedding occurs at or just before the onset of symptoms (Zou et al., 2020), so it is common for asymptomatic people to unknowingly transmit the disease and those patients may have CT opacities (Varble et al., 2021). CT should not replace RT-PCR for the diagnosis of SARS-CoV-2/COVID-19, so its use might be tightly linked to RT-PCR availability, and the risk-to-benefit ratios of the radiation risk vs. the risk to the population of not diagnosing or isolating a particular patient. CT might also be proposed as an epidemiology tool to assess spread or focus on a target population who has exposure history, such as a cruise ship.

### Precedent

There is a precedent for widening the role of chest CT in COVID-19 evaluation. Although some US and UK guidelines have recommended against using chest CT for screening (NHCotPsRo, 2019; Long et al., 2020), other countries have applied chest CT to specific outbreak settings with a more widespread, targeted, and strategic use of chest CT. In Chinese outbreak settings, for example, chest CT of high-risk populations, alongside of strict public health containment strategies, may have contributed to successful containment and low mortalities. In the US and Europe, chest CT has mainly been used to examine patients later in the disease cycle, such as to screen for complications or superinfections. In contrast, chest CT was used frequently along with RT-PCR for acute diagnosis in “fever clinics” in Hubei Province, China (Liang, 2020). For a brief and transient period during a high outbreak setting, COVID-19 diagnoses were made based on positive CT and a recent high-risk travel or possible exposure, even when the patients’ RT-PCR results were initially negative (NHCotPsRo, 2019). Thus, CT has been aggressively applied alongside of RT-PCR in an outbreak setting as an epidemiology tool for optimized contact tracing.

Furthermore, early in the pandemic, the United Kingdom provided intercollegiate guidelines stating that patients undergoing elective surgery required chest CT beforehand if the procedures were high risk or if the patients were expected to require admission to the intensive therapy unit or high dependency unit postoperatively (Standards and Clinical Gu, 2020). Additionally, for patients presenting with abdominal symptoms and undergoing CT evaluation of the abdomen and pelvis, CT coverage of the chest also was recommended early in the pandemic (Bullock et al., 2020).

### Potential Roles of Artificial Intelligence in CT Optimization

**FIGURE 1:**
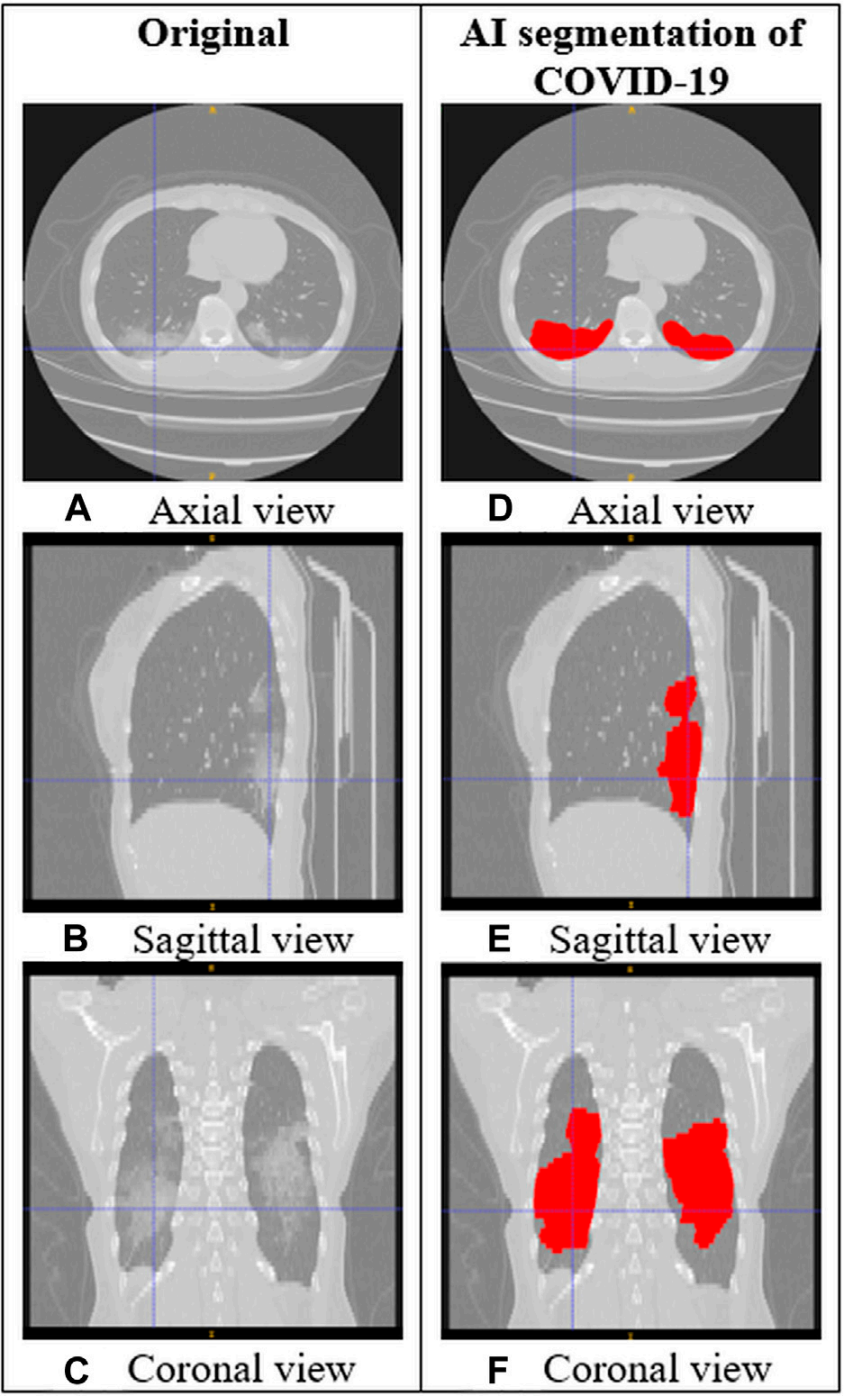
CT images of the lungs in a subject with COVID-19. **(A–C)** Original CT images. **(D–F)** CT images with AI segmentation of COVID-19 lung opacities.

AI is a promising tool to increase the potential suitability of chest CT for COVID-19 screening, detection, monitoring, and research in certain highly constrained settings. AI has already been applied in many technologies developed in response to the emergence of COVID-19 (Bullock et al., 2020), including lung and infection segmentation, COVID-19 quantification, and techniques for clinical classification, detection, assessment, and monitoring. Figure 1 shows an example of how AI segmentation models for COVID-19 can be applied to chest CT.

Currently, a common recommendation among medical and public health organizations is that CT should not be used for screening, and it should only be used for COVID-19 in patients with moderate to severe symptoms of COVID-19, worsening respiratory status, or indications of cardiopulmonary complications, and only when RT-PCR is unavailable or RT-PCR access is highly limited. Despite the high sensitivity of chest CT for COVID-19 in high-prevalence settings, one reason why chest CT is not more widely recommended for COVID-19 diagnosis is that the imaging findings are nonspecific, overlapping with many other diseases such as influenza, H1N1, SARS, MERS, and pneumonias with underlying causes other than SARS-CoV-2 (A. C. O. Radiology, 2020; C. F. D. C. A. Prevention, 2020). One study found that two standardized grading systems for determining the suspicion level of COVID-19 pneumonia based on chest CT had good inter-reader agreement among experienced readers but only moderate inter-reader agreement among less experienced readers (Sushentsev et al., 2020). To increase specificity and reduce the analytical burden on radiologists, AI could be utilized to learn the unique characteristics of chest CT in COVID-19 compared to other diseases (Roberts et al., 2021). This would be especially useful for smaller medical centers that lack dedicated, experienced radiologists and for out-of-hours scanning. Standardized AI initial interpretations might also reduce the subjectivity or interobserver variability of a subsequent radiologist review.

Lin et al. (2021) investigated differences in CT characteristics, some of which were determined by AI software, between patients with COVID-19 pneumonia and influenza virus pneumonia. Although they identified four CT characteristics with statistical differences, they concluded it might still be difficult to differentiate between the two causes of pneumonia in clinical practice.

In a particularly promising study by Harmon et al. (2020), a series of deep learning algorithms were used to assist chest CT in distinguishing between COVID-19 pneumonia and non-COVID-19 pneumonia. The algorithms were trained in a cohort of 1,280 patients and tested in an independent set of 1,337 patients. The resulting classification of COVID-19 pneumonia had an accuracy of >90%, sensitivity of 84%, and specificity of 93%. Among 140 patients with laboratory confirmed non-COVID-19 pneumonia in the test set, the false positive rate of the AI-assisted chest CT was 10%.

However, the overall pool of research on AI-assisted chest CT is at a very early stage, with many limitations, generalizations, overfit models, and reproduction of similar models. Image-based AI models abound that lack external validation, clinical metadata and relevant clinical labs, phase of disease parameters, or background prevalence data. Two separate reviews on this research topic have highlighted the shortcomings. One was a systematic review on machine learning literature related to the use of CT and CXR imaging for COVID-19 diagnosis and prognosis (Roberts et al., 2021). It did not find any articles that satisfied all three key criteria for clinical translation of research: sufficiently documented and reproducible methods, methods adhering to best practices for machine learning model development, and proper external validation. The other review was on the use of AI with chest CT to diagnose COVID-19 (Ozsahin et al., 2020). It found that the majority of studies on this topic were not peer-reviewed. Additionally, some studies had extremely limited data and some used data from different institutions and scanners, but did not carry out the necessary preprocessing of the data to increase the consistency across data from different sources. Furthermore, some studies lacked sufficient demographic or clinical information for the patients. The two review articles agree that larger and higher quality datasets will be of the utmost importance for future studies in the field. COVID-Net aims to accelerate research in the field by releasing open-source, open-access deep learning models and networks created for AI-assisted chest CT for various COVID-19 related tasks (e.g., screening and treatment monitoring) as well as large, diverse datasets (Wong, 2020). Federated learning incentivizes and facilitates data sharing by removing certain privacy barriers, which have variable rules and processes throughout the world. With federated learning, the deep learning model weights can be shared without actual data exchange, without compromising the AI training process (Yang et al., 2021).

One of the risks associated with CT imaging is radiation exposure. Regarding patient safety, AI-based estimations of body region thickness could calculate optimal X-ray exposure parameters and AI-generalized adversarial networks could facilitate low-dose imaging. The population risks of more broadly using chest CT scans for enhanced indications in COVID-19 remain to be defined or fully justified for specific settings and remain a concern.

Looking forward, AI could be used to improve the sensitivity and efficiency of chest CT for detecting COVID-19 and to increase the effectiveness of CT as a disease response or a monitoring tool for both research and clinical practice. AI could further enhance the ability of CT to provide quantification of objective biomarkers of response to experimental treatment programs for specific cohorts of patients. AI could also help optimize the CT image quality through the development of automated, high-precision Iso-centering and scan range determination.

CT AI might provide a standardized measure and surrogate biomarker metric for lung disease. Speculative uses of CT AI include the measurement of clinical response to therapeutics like steroids, monoclonal antibodies, or anti-inflammatory medications. It is unknown whether CT AI could help identify chronic lung changes in chronic COVID-19 breathlessness, variant-specific features, or early detection of at-risk phases of disease exacerbations (either primary infectious opacities or immune or inflammatory phase reactions). We have a lot to learn and CT AI adds a tool to the discovery toolbox.

## Conclusion

In conclusion, there are compelling reasons to be attracted toward studying chest CT as a tool for developing a better understanding of COVID-19. Clinical public health and research goals for COVID-19 might be enlightened by a targeted application of chest CT and CT AI to seeking answers for specific clinical and research questions in specific outbreak or therapeutic settings. Additional research and data are required to determine the exact roles of chest CT in early detection, classification, and triage of resource-limited therapies, and its potential role as an epidemiology tool in limited targeted populations. The pandemic has proved to be an “AI-instigator”; however, AI-assisted CT tools will require more rigorous academic development, broader clinical metadata, and more thorough critical review in hopes of more generalized tools with clinical or research relevance. We can do better. The times demand it.

## Data Availability

The original contributions presented in the study are included in the article; further inquiries can be directed to the corresponding author.

## Disclaimer

The content of this manuscript does not necessarily reflect the views, policies, or opinions of the U.S. Department of Health and Human Services nor the National Institutes of Health. The mention of commercial products, their source, or their use in connection with material reported herein is not to be construed as an actual or implied endorsement of such products by the United States government. Opinions expressed are those of the authors, not necessarily the NIH. NIH has a cooperative research and development agreement with Philips, Siemens, and Canon Medical. Canon Medical and NIH have a licensing agreement for a software algorithm related to classification of COVID-19 on chest CT.
